# Associations of osteoporosis and sarcopenia with frailty and multimorbidity among participants of the Hertfordshire Cohort Study

**DOI:** 10.1002/jcsm.12870

**Published:** 2021-12-06

**Authors:** Faidra Laskou, Nicholas R. Fuggle, Harnish P. Patel, Karen Jameson, Cyrus Cooper, Elaine Dennison

**Affiliations:** ^1^ Medical Research Council Lifecourse Epidemiology Centre University of Southampton Southampton UK; ^2^ NIHR Southampton Biomedical Research Centre University of Southampton and University Hospitals Southampton NHS Foundation Trust Southampton UK; ^3^ The Alan Turing Institute London UK; ^4^ Medicine for Older People University Hospital Southampton Southampton UK; ^5^ Academic Geriatric Medicine University of Southampton Southampton UK; ^6^ NIHR Oxford Biomedical Research Unit University of Oxford Oxford UK; ^7^ Victoria University of Wellington Wellington New Zealand

**Keywords:** Sarcopenia, Osteoporosis, Osteosarcopenia, Frailty, Multimorbidity, Prevalence

## Abstract

**Background:**

Ageing is commonly associated with sarcopenia (SP) and osteoporosis (OP), both of which are associated with disability, impaired quality of life, and mortality. The aims of this study were to explore the relationships between SP, OP, frailty, and multimorbidity in community‐dwelling older adults participating in the Hertfordshire Cohort Study (HCS) and to determine whether coexistence of OP and SP was associated with a significantly heavier health burden.

**Methods:**

At baseline, 405 participants self‐reported their comorbidities. Cut‐offs for low grip strength and appendicular lean mass index were used according to the EWSGOP2 criteria to define SP. OP was diagnosed when *T*‐scores of < −2.5 were present at the femoral neck or the participant reported use of the anti‐OP medications including hormone replacement therapy (HRT), raloxifene, or bisphosphonates. Frailty was defined using the standard Fried definition.

**Results:**

One hundred ninety‐nine men and 206 women were included in the study. Baseline median (interquartile range) age of participants was 75.5 (73.4–77.9) years. Twenty‐six (8%) and 66 (21.4%) of the participants had SP and OP, respectively. Eighty‐three (20.5%) reported three or more comorbidities. The prevalence of pre‐frailty and frailty in the study sample was 57.5% and 8.1%, respectively. Having SP only was strongly associated with frailty [odds ratio (OR) 8.28, 95% confidence interval (CI) 1.27, 54.03; *P* = 0.027] while the association between having OP alone and frailty was weaker (OR 2.57, 95% CI 0.61, 10.78; *P* = 0.196). The likelihood of being frail was substantially higher in the presence of coexisting SP and OP (OR 26.15, 95% CI 3.13, 218.76; *P* = 0.003). SP alone and OP alone were both associated with having three or more comorbidities (OR 4.71, 95% CI 1.50, 14.76; *P* = 0.008 and OR 2.86, 95% CI 1.32, 6.22; *P* = 0.008, respectively) although the coexistence of SP and OP was not significantly associated with multimorbidity (OR 3.45, 95% CI 0.59, 20.26; *P* = 0.171).

**Conclusions:**

Individuals living with frailty were often osteosarcopenic. Multimorbidity was common in individuals with either SP or OP. Early identification of SP and OP not only allows implementation of treatment strategies but also presents an opportunity to mitigate frailty risk.

## Introduction

Sarcopenia (SP), osteoporosis (OP), and frailty are highly prevalent in older adults but are frequently under‐recognized. They have all been shown to have an adverse impact on quality of life and are associated with disability and mortality.^1^ United Nations estimations showed that in 2010 there were 524 million people in the world aged 65 years old and over, with projections indicating that this number will increase to 1.5 billion by 2050 (a three‐fold increase).^2^ Hence, identification of individuals who might be particularly vulnerable to the adverse outcomes of musculoskeletal ageing is of clinical and public health concern.

Sarcopenia is characterized by progressive and generalized decline in muscle strength, function, and muscle mass with increasing age or secondary to a disease process. SP increases the likelihood of falls and adversely impacts on functional independence and quality of life.^3^, ^4^ OP, the commonest metabolic bone disease in older people, is characterized by both low bone mass and microarchitectural deterioration that predisposes to low‐energy transfer fragility fractures. These are associated with chronic pain, impaired physical function, loss of independence, and a higher risk of short‐term and longer‐term institutionalization.^5^ Consequently, both these conditions confer a high health burden for the individual as well as health care systems. ‘Osteosarcopenia’ is the term given when OP and SP occur in consort and recent intense focus has been on their combined effects on current and future health.

The syndrome of frailty is associated with, but not an inevitable consequence of ageing and is characterized by a vulnerability to stressor events that can be both internal and external.^6^ Both frailty and pre‐frailty, the prodromal state before the onset of clinically identifiable frailty, are associated with adverse outcomes.^7^ The most widely used definitions of physical frailty are the phenotype model described by Fried, where frailty is identified by the presence of at least three out of five physical characteristics: weight loss, exhaustion, low energy expenditure, slow walking speed, and low handgrip strength.^8^ The cumulative deficit model of frailty described by Rockwood *et al*. also predicts adverse health outcomes and comprises age‐associated accumulation of deficits that range from symptoms, sensory deficits, clinical signs, diseases, disabilities, and abnormal laboratory test results.^9^

Few other studies have considered interrelationships between SP, OP, and frailty. For example, participants with SP were reported to have a high incidence of OP, a higher incidence of falls and fractures,^10^, ^11^, ^19^ but in these analyses frailty was not considered the outcome. For instance, patients with SP had 12.9 times higher odds of having OP and 2.7 times higher odds of having fractures than the non‐sarcopenic ones in a population‐based Finnish study,^20^ and similarly, bone mineral density (BMD) was found to be lower in sarcopenic individuals in the Copenhagen Sarcopenia study, increasing the risk of having OP.^21^ Older males with a diagnosis of probable and definite SP were eight times more likely to have a diagnosis of osteopenia or OP,^22^ where in postmenopausal Brazilian women, SP and severe SP was shown to impose a higher risk for OP adding to the growing evidence that SP and OP frequently co‐occur.^23^

However, studies that have considered frailty as an outcome suggest that the risks of serious morbidity are notably higher when OP and SP coexist.^24^, ^26^ Individuals with osteosarcopenia have also increasingly higher risk of mortality compared with those with SP or OP alone.^27^

Given that ageing is commonly associated with SP and OP, the aims of this study were (1) to explore associations between SP and OP, individually or in combination, with frailty in community‐dwelling older adults participating in the Hertfordshire Cohort Study (HCS), and (2) to determine if coexistence of both SP and OP (osteosarcopenia) carries a higher likelihood of being frail. Given the importance of multimorbidity on health outcomes,^28^ and the association that previously has been described between osteosarcopenia and chronic diseases,^29^ we also considered (3) whether the coexistence of SP and OP was associated with a significantly heavier health burden, as assessed by the number of concurrent long‐term conditions. The wealth of phenotypic information collected in the HCS, a cohort study of community‐dwelling older adults, has allowed us to describe the prevalence and pre‐frailty in this group and to consider whether the coexistence of SP and OP in individuals interacted to amplitude risk of frailty. This is of high clinical relevance as the identification of coexistent SP and OP coexistence not only allows early treatment and management strategies but might also offer an opportunity to mitigate frailty risk.^5^, ^6^

## Methods

### Study participants

The HCS was designed to examine the relationship between growth in infancy and the subsequent risk of common adult diseases, including OP and SP, and has been described in detail elsewhere.^30^ Participants have been followed up at a number of time points since its inception. The present study was performed using baseline data collected in 2011. All study participants provided written informed consent, and ethical approval was obtained from the Hertfordshire Research Ethics Committee. All participants gave written informed consent.

### Data collection

#### Questionnaire and anthropometry

Participants completed questionnaires that comprised questions related to lifestyle including smoking habits, alcohol consumption, physical activity (LASA Physical Activity Questionnaire—LAPAQ), and nutrition (Short Food Frequency Questionnaire—FFQ). Anthropometric measurements including height and weight were measured to calculate body mass index (BMI).

#### Physical performance and muscle mass

Grip strength was measured three times in each hand using a Jamar hand‐held isokinetic dynamometer using a standardized protocol.^31^ The maximum value was used in analyses. Gait speed (metres per second) determined after timed 8 foot walk test. The use of assistive devices was permitted, if required. For chair rises (also used to assess for physical function), the time taken for participants to stand up and sit down again (with their arms crossed across their chest) a total of five times was recorded.

Skeletal muscle mass was measured with a body composition dual‐energy X‐ray absorptiometry (DXA) scan (Lunar Prodigy Advanced) to quantify regional as well as total lean mass, fat mass, and bone mineral content. Proximal femur BMD values were determined using standard DXA.

### Definitions of sarcopenia, osteoporosis, frailty, and comorbidity

#### Sarcopenia

Sarcopenia was defined using the revised EWSGOP2 criteria for low muscle strength measured by hand grip strength (<27 kg in men and <16 kg in women) or slow chair rise time (>15 s for five rises) and low muscle quantity [appendicular skeletal mass (ASM) index (ASM/height^2^) < 7.0 kg/m^2^ in men and <5.5 kg/m^2^ in women].^32^

#### Osteoporosis

Osteoporosis was defined according to the World Health Organization criteria and diagnosed when BMD *T*‐scores were lower than the peak bone mass by 2.5 SD at the femoral neck or the use of osteoporotic treatment including hormone replacement therapy (HRT), bisphosphonates, or raloxifene was reported.

#### Frailty

Frailty was defined using the standard Fried definition using similar cut‐offs for muscle strength that were used to define SP (grip strength <27 kg in men and <16 kg in women) in addition to the presence of unintentional weight loss, self‐reported exhaustion, and lowest sex‐specific fifth of activity time: 0 out of 5 domains = non‐frail, 1 or 2 domains = pre‐frail, and ≥3 domains = frail. The presence of unintentional weight loss was defined as a positive answer to the question: ‘In the last year, have you lost more than 10 pounds (4.5 kg) unintentionally (i.e. not due to dieting or exercise)?’. The presence of self‐reported exhaustion was defined as an answer of a moderate amount of time or most of the time (i.e. ≥3 days) to the question: How often in the last week did you feel ‘everything I did was an effort’ or ‘I could not get going’.

#### Comorbidity

Participants were asked to self‐report their comorbidities with the use of a questionnaire. We then categorized the number as none, one, two, and three or more. Multimorbidity was defined when participants self‐reported three or more comorbidities.

### Statistical analysis

Descriptive statistics for continuous variables were expressed as mean and standard deviation (SD) or median and interquartile range (IQR) as appropriate. Categorical variables were expressed as frequency (*N*) and percentage (%). Differences between groups (such as frailty status) were assessed using analysis of variance (ANOVA), Kruskal–Wallis tests, Pearson's *χ* ^2^ tests, or Fisher's exact tests as appropriate. Logistic regressions were performed to analyse associations of OP at femoral neck, SP, and coexistence of OP and SP as explanatory variables for frailty adjusting for sex only initially, then further adjusting for age, BMI, current smoker, and alcohol consumption.

## Results

Complete baseline data were available for 405 participants (199 men and 206 women). The median (SD) age of participants at baseline was 75.5 (73.4–77.92) years. The characteristics of this population are shown in *Table* 1; 4.5% of men and 2.9% of women were current smokers with no sex difference (*P* = 0.391). There was a significant difference between women and men at baseline in the median (IQR) alcohol consumption [men: 6.2 (1.0–12.3) units per week; women: 0.3 (0.0–3.1) units per week; *P* < 0.001]. Over half of participants reported to have low walking speed (≤0.8 m/s) (55.2%, 217/393) and one‐fifth of them had low physical activity (80/394). One‐fifth of participants had evidence of OP and 8% had evidence of SP; 20.5% (83/405) self‐reported three or more comorbidities.

**Table 1:** Baseline characteristics of all participants

	All participants		
	*N*	Median	IQR
Age (years)	405	75.5	73.4–77.9
Weight (kg)	405	76.0	68.2–85.8
BMI (kg/m^2^)	402	27.4	25.1–30.9
Alcohol consumption (units per week)	405	2.0	0.1–8.3
Activity time in the last 2 weeks (min/day)	394	190	124–283

	*N*	Mean	SD
Height (cm)	402	165.7	9.3
Maximum grip (kg)	405	28.7	10
Gait speed (m/s)	393	0.75	0.18
ALM index (kg/m^2^)^a^	295	7.27	1.08
Femoral neck BMD (g/cm^2^)^b^	306	0.889	0.139

	Total *N*	*N*	%
Female sex	405	206	50.9
Unintentional weight loss	404	16	4.0
Self‐reported exhaustion	405	48	11.9
Low grip strength (<27 kg men; <16 kg women)	405	47	11.6
Low walking speed (≤0.8 m/s)	393	217	55.2
Low physical activity^c^	394	80	20.3
Emaciated (BMI < 18.5 kg/m^2^)	402	2	0.5
Current smoker	405	15	3.7
Osteoporosis^d^	308	66	21.4
Sarcopenia	323	26	8.0
Having 3 or more comorbidities	405	83	20.5

BMI, body mass index; IQR, interquartile range; SD, standard deviation.

^a^ ALM = appendicular lean mass.

^b^ Minimum of left and right femoral neck bone mineral density.

^c^ Low physical activity = lowest 20% of activity time.

^d^ Osteoporosis = femoral neck *t*‐score < −2.5 or taking hormone replacement therapy, bisphosphonates, or raloxifene.

There were significant differences between non‐frail, pre‐frail, and frail participants with respect to age (*P* = 0.002), height (*P* = 0.035), BMI (kg/m^2^) (*P* < 0.001), alcohol consumption (*P* = 0.027), physical activity in the last 2 weeks (*P* < 0.001), walking speed (*P* < 0.001), and grip strength (*P* < 0.001) as shown in *Table* 2. Of the five Fried frailty components, low walking speed and low physical activity followed by self‐reported exhaustion were the most prevalent (96.6%, 87.5%, and 75.8%, respectively) among frail participants.

**Table 2:** Characteristics of individuals by frailty category; frailty components and association with sarcopenia and osteoporosis categories

	Non‐frail			Pre‐frail			Frail			
	*N*	Median	IQR	*N*	Median	IQR	*N*	Median	IQR	*P*‐value^a^
Age (years)	139	74.7	73.1–76.8	233	75.7	73.7–78.5	33	77.0	74.7–78.9	0.002*
Weight (kg)	139	74.3	68.2–81.9	233	76.6	68.2–88.0	33	78.5	70.6–88.7	0.143
BMI (kg/m^2^)	139	26.5	24.4–29.2	230	28.4	25.6–31.2	33	29.4	25.4–32.8	<0.001*
Alcohol consumption (units per week)	139	3.3	0.3–10.0	233	2.0	0.1–7.5	33	0.5	0.0–5.0	0.027*
Activity time in the last 2 weeks (min/day)	139	231	178–327	223	176	107–240	32	63	42–91	<0.001*

	*N*	Mean	SD	*N*	Mean	SD	*N*	Mean	SD	*P*‐value^a^
Height (cm)	139	167.3	9.1	230	165.0	9.3	33	163.9	9.9	0.035*
Maximum grip (kg)	139	32.4	9.3	233	27.7	9.5	33	20.6	10.1	<0.001*
Gait speed (m/s)	139	0.90	0.1	225	0.69	0.15	29	0.53	0.14	<0.001*
ALM index (kg/m^2^)^b^	110	7.28	1.08	168	7.28	1.08	17	7.08	1.10	0.745
Femoral neck BMD (g/cm^2^)^c^	115	0.892	0.127	172	0.892	0.148	19	0.853	0.134	0.500

	Total *N*	*N*	%	Total *N*	*N*	%	Total *N*	*N*	%	*P*‐value^a^
Female sex	139	62	44.6	233	125	53.6	33	19	57.6	0.174
Unintentional weight loss	139	0	0	232	8	3.4	33	8	24.2	<0.001*
Self‐reported exhaustion	139	0	0	233	23	9.9	33	25	75.8	<0.001*
Low grip strength (<27 kg men; <16 kg women)	139	0	0	233	27	11.6	33	20	60.6	<0.001*
Low walking speed (≤0.8 m/s)	139	0	0	225	189	84.0	29	28	96.6	<0.001*
Low physical activity^d^	139	0	0	223	52	23.3	32	28	87.5	<0.001*
Emaciated (BMI < 18.5 kg/m^2^)	139	2	1.4	230	0	0	33	0	0	0.277
Current smoker	139	2	1.4	233	12	5.2	33	1	3.0	0.169
Osteoporosis^e^	118	24	20.3	173	36	20.8	17	6	35.3	0.375
Sarcopenia	126	5	4.0	179	16	8.9	18	5	27.8	0.005*
Having 3 or more comorbidities	139	19	13.7	233	48	20.6	33	16	48.5	<0.001*

BMI, body mass index; IQR, interquartile range; *N*, number of participants; SD, standard deviation.

^a^ *P*‐value for the difference between the frailty categories.

^b^ ALM = appendicular lean mass.

^c^ Minimum of left and right femoral neck bone mineral density.

^d^ Low physical activity = lowest 20% of activity time as measured by LAPAQ.

^e^ Osteoporosis = femoral neck *t*‐score < −2.5 or taking hormone replacement therapy, bisphosphonates, or raloxifene.

* *P*‐value < 0.05.

**Figure 1:**
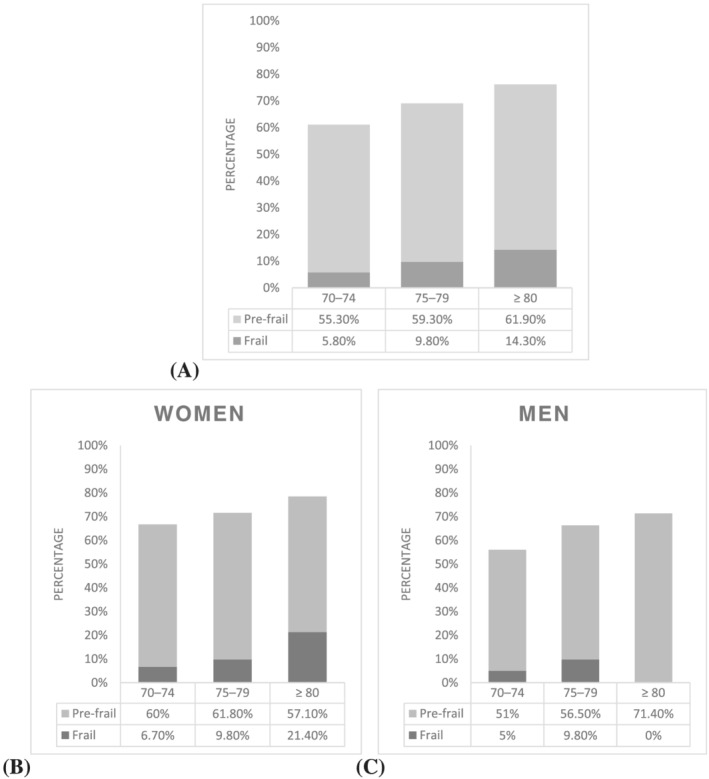
Pooled prevalence of frailty and pre‐frailty at baseline (*A*) and age‐stratified and gender‐stratified prevalence of frailty and pre‐frailty at baseline (*B* and *C*).

In our sample, the prevalence of frailty and pre‐frailty at baseline was 8.1% (men 7.0%, women 9.2%) and 57.5% (men 54.3%, women 60.7%), respectively. *Figure* 1 illustrates the prevalence of frailty in the 70–74, 75–79, and ≥80 year age groups. These were 5.8%, 9.8%, and 14.3%, respectively, and that of pre‐frailty was 55.3%, 59.3%, and 61.9%, respectively. *Figure* 1B and 1C also shows the age‐stratified and gender‐stratified prevalence of frailty and pre‐frailty. There were no significant differences in frailty status between men and women nor between the age groups in both sexes.

**Figure 2:**
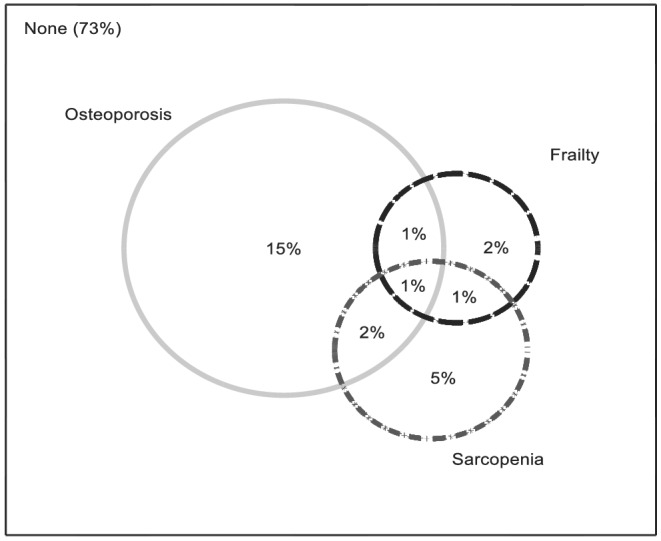
Venn diagram illustrating the relationships of osteoporosis, sarcopenia, and frailty at baseline.

We next considered interrelationships between SP, OP, or both with frailty. Coexistence of SP, OP, and frailty was observed in 1% of this population. Two per cent of the study sample had SP and OP. Seventy‐three per cent had no evidence of SP, OP, or frailty (*Figure* 2). Among the participants with frailty, 27.8% had a concomitant diagnosis of SP, compared with 8.9% in the pre‐frail and 4.0% in the non‐frail categories (*P* = 0.005). In a model of SP/OP status, having SP only was strongly associated with frailty [odds ratio (OR) 8.28, 95% confidence interval (CI) 1.27, 54.03; *P* = 0.027] while the association between having OP alone and frailty was weaker (OR 2.57, 95% CI 0.61, 10.78; *P* = 0.196). The likelihood of being frail was substantially higher in the presence of coexisting SP and OP (OR 26.15, 95% CI 3.13, 218.76; *P* = 0.003) (*Table* 3). Having SP alone and OP alone were both associated with having three or more comorbidities (OR 4.71, 95% CI 1.50, 14.76; *P* = 0.008 and OR 2.86, 95% CI 1.32, 6.22; *P* = 0.008, respectively) although this relationship was not stronger with coexisting SP and OP (OR 3.45, 95% CI 0.59, 20.26; *P* = 0.171) (*Table* 4).

**Table 3:** Relationship of sarcopenia, osteoporosis, and frailty at baseline

	Adjusted for sex only				Fully adjusted			
	*N*	OR	95% CI	*P*‐value	*N*	OR	95% CI	*P*‐value
OP and SP status	293				284			
Neither OP nor SP (reference)								
OP only		2.24	0.53, 9.56	0.274		2.57	0.61, 10.78	0.196
SP only		3.77	0.68, 20.90	0.129		8.28	1.27, 54.03	0.027*
Both OP and SP		9.98	1.60, 62.22	0.014*		26.15	3.13, 218.76	0.003*

CI, confidence interval; *N*, number of participants; OP, osteoporosis; OR, odds ratio; SP, sarcopenia.

Values in the fully adjusted categories are adjusted for sex, age, body mass index, current smoking history, and alcohol consumption. OP values refer to a femoral neck *T*‐score of < −2.5 or taking hormone replacement therapy, bisphosphonates, or raloxifene. SP was defined using the EWSGOP2 criteria.

* *P*‐value < 0.05.

**Table 4:** Relationship of sarcopenia, osteoporosis, and multimorbidity (≥3 comorbidities) at baseline

	Adjusted for sex only				Fully adjusted			
	*N*	OR	95% CI	*P*‐value	*N*	OR	95% CI	*P*‐value
OP and SP status	288				286			
Neither OP nor SP (reference)								
OP only		2.76	1.35, 5.67	0.006*		2.86	1.32, 6.22	0.008*
SP only		2.98	1.02, 8.74	0.047*		4.71	1.50, 14.76	0.008*
Both OP and SP		2.12	0.39, 11.64	0.385		3.45	0.59, 20.26	0.171

CI, confidence interval; *N*, number of participants; OP, osteoporosis; OR, odds ratio; SP, sarcopenia.

Values in the fully adjusted categories are adjusted for sex, age, body mass index, current smoking history, and alcohol consumption. OP values refer to a femoral neck *T*‐score of < −2.5 or taking hormone replacement therapy, bisphosphonates, or raloxifene. Comorbidities self‐reported by participants. SP was defined using the EWSGOP2 criteria.

* *P*‐value < 0.05.

## Discussion

In this study, we examined the association between SP, OP, or coexistence of SP and OP, with frailty and multimorbidity in 405 community‐dwelling older men and women. We also report prevalence and incidence of frailty in the same group of community‐dwelling older adults. As might be anticipated, SP was associated with frailty, but the association of OP with frailty was weaker. However, we also reported that the likelihood of being frail was markedly higher in the presence of coexisting SP and OP than with SP alone. We had anticipated relationships between SP and frailty because of the diagnostic criteria for the two conditions.^33^ For this reason, we also considered relationship with multimorbidity as a proxy marker for frailty. Both SP and OP were associated with multimorbidity, but in this case there did not appear to be an interaction between the two conditions.

The concept of osteosarcopenia is relatively new, but in previous work coexistence of SP and OP has been associated cross‐sectionally with depression, malnutrition, peptic ulcer disease, inflammatory arthritis, and reduced mobility.^34^ Studies from Australia and China have demonstrated that individuals with both OP and SP are at higher risk of falls and fractures than those with OP or SP alone.^24^, ^34^ Only a few studies have examined the association between both OP and SP with frailty in community‐dwelling older adults. In the Women's Health and Aging Studies II, the likelihood of being frail was higher in the presence of these two conditions, but the association was not statistically significant. The criteria used in this study to assess SP was appendicular lean mass by height^2^ without taking into account muscle strength, possibly leading to an under‐recognition of sarcopenic participants.^35^ In the SARCOS study, low lean mass together with OP showed an association with frailty; cut‐offs for lean mass were based on FNIH criteria, and the authors aimed to characterize the phenotype of sarcopenic older adults only based on lean mass.^36^ In a study of octogenarians in China, women were more likely to have osteosarcopenia compared with men; SP and OP alone or in combination were associated with frailty.^24^ OP, risk of falls, and SP were reported in the I‐Lan Longitudinal Aging Study to be associated with frailty independently.^37^ In a hospital‐based study, SP was strongly associated with frailty (*P* < 0.001) while relationships with OP were weaker (*P* = 0.055).^38^ Finally, in a retrospective study among postmenopausal patients with known OP attending a hospital bone clinic, those with a diagnosis of osteosarcopenia were more likely to be frail than those with OP alone.^25^

As expected, frailty and pre‐frailty were more prevalent in individuals over the age of 80 in both sexes. Both the prevalence of frailty and pre‐frailty were increased with age in women, but only pre‐frailty was increased with age in men; however, the number of men aged over 80 in our study was low. Previous UK‐based studies report similar prevalence of frailty to our study, but sex differences were noted.^39^ Weighted prevalence in the English Longitudinal Study of Ageing (ELSA) study was 14% among participants age 60–90 years^40^; prevalence did increase with age, was more common in women, and was associated with a burden in regard to mobility, and everyday activities.

Other groups studying an older population have found the prevalence of frailty and pre‐frailty to be similar to our study,^37^, ^41^, ^43^ although different population sampling and definitional approaches may lead to differences in findings. For example, in a community‐dwelling cohort of men and women in Japan with mean age of 70.3 (SD 11.0) years, the prevalence of frailty was estimated to be 5.6% in both sexes (in the same cohort, frailty was more common in the presence of both SP and OP).^44^ In a recent systematic review and meta‐analysis, the pooled prevalence of frailty for community dwellers aged ≥50 years, using full and recognized modifications of Fried's criteria, was 12% across 62 counties worldwide compared with 24% when other measures of frailty were used, highlighting that instrument selection influences prevalence proportions. In Europe specifically, the prevalence of frailty was 8%, a percentage close to our calculated prevalence, when using physical frailty measures and that of pre‐frailty was 42%.^45^ Far fewer data are available regarding the prevalence of pre‐frailty in community‐dwelling populations. The Survey of Health, Aging and Retirement in Europe (SHARE) was one of the few studies assessing pre‐frailty and found that the prevalence of pre‐frailty in individuals over the age of 50 in 10 European countries ranged between 34.6% and 50.9%.^46^ In our study, the prevalence of pre‐frailty was higher, ranging from 55.3% to 61.9%. Approximately 43.4 and 150.6 per 1000 person‐years was the estimated incidence of frailty and pre‐frailty, respectively, in a recent systematic review and meta‐analysis,^47^ a higher incidence compared with our study although there is likely to be substantial geographic variation when measuring incidence.^48^

Our study has some limitations. Participants in the HCS cohort study are all community living and therefore might be expected to show a healthy cohort bias, limiting our ability to discern some relationships. This is reflected by the relatively low number of sarcopenic and frail individuals and by the fact that participants for whom data were available at follow‐up and developed frailty were overall healthier. They were younger, heavier, taller, stronger, and faster walkers and had better physical activity compared with those for whom data were not available on incidence frailty. However, this study has been able to draw on the detailed phenotypic information available in the HCS to report the epidemiology of coexisting OP and SP, and its association with multimorbidity and frailty. Furthermore, HCS participants have been compared with those in the nationally representative Health Survey for England and have been found to be broadly comparable in terms of their health and lifestyle. We therefore suggest that the results from the current study could be reasonably generalized to the wider population of older men and women.

Our study suggests that the likelihood of being frail was markedly higher when SP and OP were coexistent. However, the relationship may be bidirectional given the risk factors and pathophysiological pathways that drive individual conditions. Future longitudinal cohort studies of older people who are diverse in both ethnicity and socio‐economic status may provide a more comprehensive understanding to the relationship between osteosarcopenia and frailty.

## Conclusions

We have shown an overall prevalence of frailty in community‐dwelling older UK adults of 8.1% with the risk increasing with age. Corresponding figures for pre‐frailty were 57.5% with the risk increasing with age only in females. We found that the presence of baseline SP and OP together are associated with a much higher risk of frailty cross‐sectionally than either condition alone and that SP and OP are both closely linked with multimorbidity. As the presence of coexisting SP and OP were highly associated with frailty, appropriate treatment and early intervention of these conditions can have a clinical benefit to reduce the progression to frailty. Furthermore, identifying and treating individuals with pre‐frailty and probable SP as early and reversible risk states may be associated with better health care outcomes and lower risk of developing frailty. Muscle and bone interrelationships need to be further studied in large prospective longitudinal cohorts as better understanding of the epidemiology of osteosarcopenia is extremely relevant to inform the development of future interventions and therapeutics to maintain older people's independence.

## Funding

F.L. and H.P.P. are supported by the NIHR Southampton Biomedical Research Centre, Nutrition, and the University of Southampton. This report is independent research, and the views expressed in this publication are those of the authors and not necessarily those of the NHS, the NIHR, or the Department of Health. These funding bodies had no role in writing of the manuscript or decision to submit for publication.

## Conflict of interest

E.D. declares consultancy and speaker fees from Pfizer, UCB, and Lilly. C.C. has received lecture fees and honoraria from Amgen, Danone, Eli Lilly, GSK, Kyowa Kirin, Medtronic, Merck, Nestlé, Novartis, Pfizer, Roche, Servier, Shire, Takeda, and UCB outside of the submitted work. N.F. declares travel bursaries from Pfizer and Eli Lilly. H.P.P. has received lecture fees from Abbott, Pfizer, and HC‐UK conferences outside of the submitted work. K.J. has nothing to declare.
